# Cardiac Tamponade

**DOI:** 10.21980/J81D1D

**Published:** 2020-10-15

**Authors:** Alan Chu, Jennifer Yee

**Affiliations:** * The Ohio State University, Department of Emergency Medicine, Columbus, OH

## Abstract

**Audience:**

This simulation is designed to educate emergency medicine residents and medical students on the recognition and management of cardiac tamponade, as well as encourage providers to become familiar with their states’ disclosure laws for sentinel events.

**Introduction:**

Cardiac tamponade is an emergent condition in which the accumulation of pericardial fluid and the consequent increase in hydrostatic pressure becomes severe enough to compromise the normal diastolic and systolic function of the heart, resulting in hemodynamic instability.^1^ The causes of cardiac tamponade are numerous because it is a potential complication of any of a number of pericardial disease processes, including infectious, inflammatory, traumatic, and malignant etiologies.^1^,^2^ Clinical presentations may vary and symptoms can be non-specific, which can lead to delayed or missed diagnoses and poor patient outcomes.^3^ In addition to this, the incidence of this condition is rising due to the increasing frequency of cardiac procedures performed (ie, pacemaker placement).^4^ Therefore, it is important for medical providers to have a high index of suspicion for the diagnosis based on patient presentation and to quickly provide necessary treatment to stabilize the patient.

**Educational Objectives:**

By the end of this simulation session, the learner will be able to: (1) describe a diagnostic differential for dizziness (2) describe the pathophysiology of cardiac tamponade (3) describe the acute management of cardiac tamponade, including fluid bolus and pericardiocentesis (4) describe the electrocardiogram (ECG) findings of pericardial effusion (5) describe the ultrasound findings of cardiac tamponade (6) describe the indications for emergent bedside pericardiocentesis versus medical stabilization and delayed pericardiocentesis for cardiac tamponade (7) describe the procedural steps for pericardiocentesis, and (8) describe your state’s laws regarding disclosure for sentinel events.

**Educational Methods:**

This session is conducted using high-fidelity simulation, followed by a debriefing session on evaluation and treatment of cardiac tamponade. However, it may also be run as an oral board case.

**Educational Methods:**

Our residents were provided an electronic survey at the completion of the debriefing session so they may rate different aspects of the simulation, as well as provide qualitative feedback on the scenario. This survey is specific to the local institution’s simulation center.

**Results:**

Feedback was largely positive because many learners mentioned during debriefing that they are not comfortable with pericardiocentesis and have limited opportunities to practice the procedure. None of our residents were familiar with our state’s or institution’s disclosure laws for sentinel events.

The local institution’s simulation center feedback form is based on the Center of Medical Simulation’s Debriefing Assessment for Simulation in Healthcare (DASH) Student Version Short Form with the inclusion of required qualitative feedback if an element was scored less than a 6 or 7.^5^ This session received a majority of 6 (consistently effective/very good) and 7 scores (extremely effective/outstanding).

**Discussion:**

This is a potential method for educating future medical providers on the diagnosis and management of cardiac tamponade in an emergency department setting. Learners initially had a wide range of differentials for the chief complaint of dizziness. We used an ECG with low voltage but without electrical alternans. When asked to provide an ECG interpretation, low voltage was intermittently explicitly interpreted by learners. We were concerned that if we showed an ECG with electrical alternans, learners may quickly arrive at the diagnosis without focusing on the subtleties of a physical exam, including looking for jugular venous distention (JVD) or pulsus paradoxus.

We did not have the patient decompensate if their international normalized ratio (INR) was not immediately reversed, given likely delay for *in vivo* coagulation to occur in the face of life-threatening tamponade, but this provided a robust discussion during debriefing if reversal should be emergently initiated.

Many residents voiced that they were uncomfortable performing a pericardiocentesis because they only had a few opportunities to do so on human cadavers, and they appreciated the opportunity to review this.

Unexpectedly, when the patient asked the learners if he should sue the cardiologist, the majority of groups told the patient that the cardiologist was not liable because tamponade is a known complication of cardiac ablation and likely reviewed this while obtaining informed consent. None of the learners were familiar with Ohio’s disclosure laws for sentinel events. This identified a gap in knowledge that may be addressed in future learning sessions.

Our main take-away is to continue providing low-frequency, high-acuity cases that provide the opportunity to review infrequent pathologies and procedures, as well as including patient safety and administrative learning points.

**Topics:**

Medical simulation, cardiac tamponade, pericardial effusion, cardiac emergencies, obstructive shock, sentinel events, iatrogenic injury, medical disclosure.

## USER GUIDE

**Table:** 

List of Resources:	
Abstract	84
User Guide	86
Instructor Materials	88
Operator Materials	97
Debriefing and Evaluation Pearls	100
Simulation Assessment	103

**Learner Audience:**

Emergency Medicine junior residents, senior residents

**Time Required for Implementation:**

Instructor Preparation: 30 minutes

Time for case: 20 minutes

Time for debriefing: 40 minutes

**Recommended Number of Learners per Instructor:**

3–4

**Topics:**

Medical simulation, cardiac tamponade, pericardial effusion, cardiac emergencies, obstructive shock, sentinel events, iatrogenic injury, medical disclosure.

**Objectives:**

By the end of this simulation session and debriefing, the learner will be able to:

1. Describe a broad differential for dizziness
2. Describe the pathophysiology of cardiac tamponade
3. Describe the acute management of cardiac tamponade, including fluid bolus and pericardiocentesis
4. Describe the ECG findings of pericardial effusion
5. Describe the ultrasound findings of cardiac tamponade
6. Describe the indications for emergent bedside pericardiocentesis versus medical stabilization and delayed pericardiocentesis for treatment of cardiac tamponade
7. Review procedural steps for pericardiocentesis
8. Describe your state’s laws regarding disclosure for sentinel events

### Linked objectives and methods

Cardiac tamponade is an uncommon emergency department (ED) diagnosis with many of the symptoms associated with it being consistent with more common diagnoses. The most important tool for diagnosis of this life-threatening condition is maintaining a high clinical suspicion. This simulation scenario allows learners to review the patient presentation and highlights the importance of obtaining pertinent past medical and surgical histories. Learners will have the opportunity to perform an initial assessment and explore the vast differential diagnosis of a critically ill patient with nonspecific complaints (objective 1), administer an intravenous (IV) fluid bolus to counteract tamponade physiology (objective 2), and perform early resuscitative measures (objective 3). Learners will need to acquire and interpret appropriate studies (ECG and ultrasound) to diagnose cardiac tamponade (objective 4 and 5). They will then need to apply appropriate clinical judgment in a resource-limited setting and perform a simulated pericardiocentesis (objectives 6 and 7). Afterwards, there will be discussion about the etiology and pathophysiology of cardiac tamponade, appropriate diagnosis and management of this condition, and review of indications and steps for pericardiocentesis (objectives 1–7). After reviewing the medical aspects of the case, there should also be a separate discussion regarding medical error disclosure and their medico-legal implications, as well as state- and institution-dependent laws and policies (objective 8).

### Recommended pre-reading for instructor

We recommend that instructors become familiar with the materials listed below under “References/suggestions for further reading.”

### Results and tips for successful implementation

This simulation was written to be performed as a high-fidelity simulation scenario but may also be used as a mock oral board case. We conducted this scenario three times for a total of eight emergency medicine residents divided into groups of two or three learners during November 2019. Interns and medical students may be provided with an ECG that shows electrical alternans, but we found using an ECG with low voltage without alternans created a slightly more difficult case. Covering the pericardiocentesis task trainer with a sheet prevented immature diagnostic closure for our learners with increased situational awareness, as well as keeping the bedside ultrasound off to the side of the room. While still ultrasound images are included here, we suggest using ultrasound videos for increased fidelity.

The local institution’s simulation center feedback form is based on the Center of Medical Simulation’s Debriefing Assessment for Simulation in Healthcare (DASH) Student Version Short Form with the inclusion of required qualitative feedback if an element was scored less than a 6 or 7.^5^ This session received all 6 (consistently effective/very good) and 7 scores (extremely effective/outstanding) with the exception of one 5 score (mostly effective/good) for the category “The instructor identified what I did well or poorly – and why.” The form also includes an area for general feedback about the case at the end.

Comments included, “This case was great! Forced you to think through a lot of different pathology and do a rare procedure,” as well as, “these cases are always quite humbling for me and I appreciate the feedback after the cases a lot. I feel much more in tune with tamponade awareness now after this case,” and “I appreciated learning what we did that changed the trajectory of our patient, and what would have yielded a different outcome.”

## Supplementary Information



## Figures and Tables

**Table:** 

#1	Complete blood count (CBC)
#2	Basic metabolic panel (BMP)
#3	Troponin
#4	Brain natriuretic peptide (BNP)
#5	Coagulation Panel with prothrombin (PT)/INR
#6	Portable chest radiograph with cardiomegaly (CXR)
#7	Electrocardiogram (ECG) with sinus tachycardia
#8	Ultrasound pericardial effusion
#9	Ultrasound plethoric Inferior Vena Cava (IVC)

**Table:** 

**Vitals:**	Heart Rate (HR) 110	Blood Pressure (BP) 100/60	Respiratory Rate (RR) 20
	Temperature (T) 98.6°F	Oxygen Saturation (O_2_Sat) 100% on room air	

**Table:** 

*Complete blood count (CBC)*	
White blood count (WBC)	9.3 ×1000/mm^3^
Hemoglobin (Hgb)	14.0 g/dL
Hematocrit (HCT)	41.7%
Platelet (Plt)	380 ×1000/mm^3^

**Table:** 

*Basic metabolic panel (BMP)*	
Sodium	140 mEq/L
Chloride	99 mEq/L
Potassium	4.0 mEq/L
Bicarbonate (HCO_3_)	22 mEq/L
Blood Urea Nitrogen (BUN)	20 mg/dL
Creatine (Cr)	0.9 mg/dL
Glucose	100 mg/dL
Calcium	8.0 mg/dL

**Table:** 

*Troponin*	0.03 ng/L

**Table:** 

*BNP*	86 pg/mL

**Table:** 

*Coagulation Panel*	
PT	22.5 sec
INR	2.1

**Figure:**
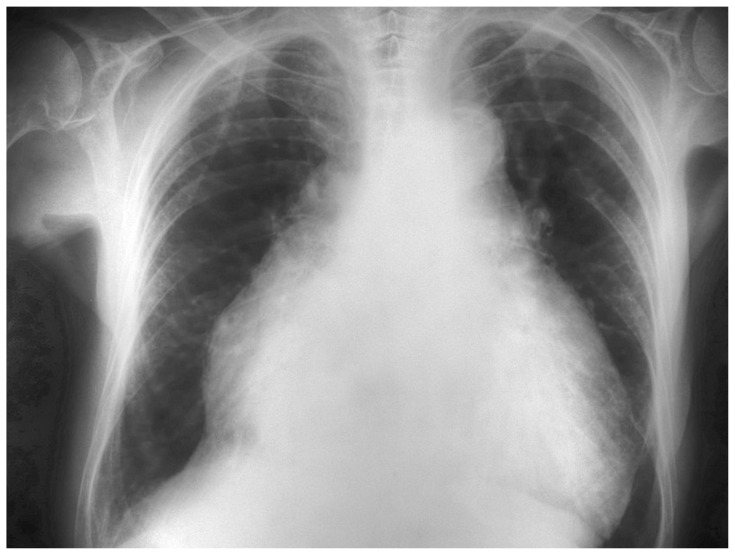


**Figure:**
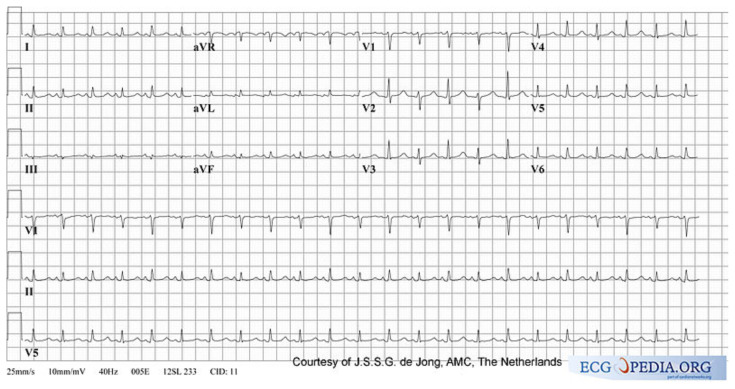


**Figure:**
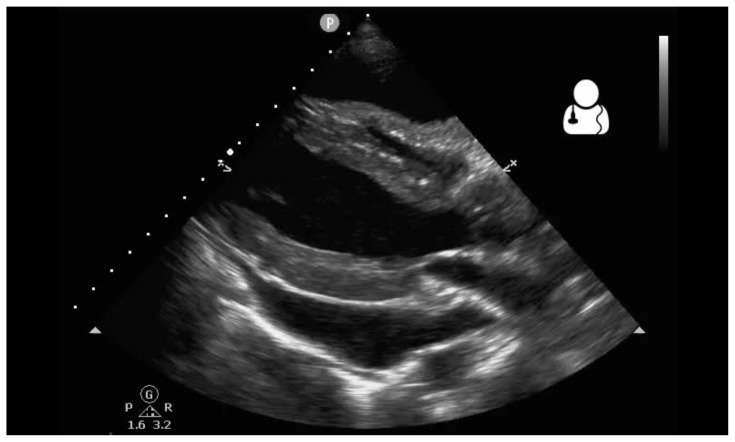


**Figure:**
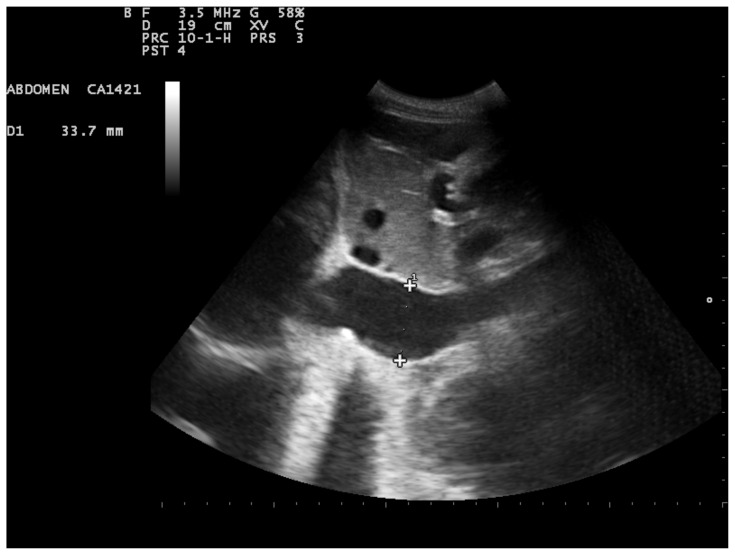


**Table:** SIMULATION EVENTS TABLE:

Minute (State)	Participant Action/Trigger	Patient Status (Simulator Response) & Operator Prompts	Monitor Display (Vital Signs)
0:00 (Baseline)	Case begins, participants place the patient on the monitor and obtain history Patient should be examined, home medications reviewed, and labs ordered.	Patient provides brief history. Will provide additional details when asked.	No vitals should be displayed until requested BP 100/60 HR 110 RR 20 O2 100% RA Temp 98.6F°
5:00	1L bolus should be ordered.	Labs should return. ECG should be provided. If IV fluid bolus is ordered, BP and heart rate will improve. (Move to **Branch Point 1**: Improvement.) If IV fluid bolus is not ordered, patient’s vitals will worsen (Move to **Branch Point 2**: Decompensation.)	With IV fluids BP 110/65 HR 100 RR 16 O2 100% RA Temp 98.6F° Without IV Fluids BP 80/50 HR 130 (sinus) RR 30 O2 97% RA Temp 98.6F
6:00 (**Branch Point 1**: Improvement)	After IV fluids ordered. Participants should recognize electrical alternans and order a bedside ultrasound.	Bedside ultrasound with cardiac tamponade should be provided. Patient should ask what’s going on, if the cardiologist messed up, and if he should sue the cardiologist.	BP 110/65 HR 100 RR 16 O2 100% RA Temp 98.6F°
8:00 (**Branch Point 1**: Improvement)	Participants should recognize cardiac tamponade on ultrasound and make decision to perform pericardiocentesis.	If participants request consultant (cardiology, cardiothoracic surgery, etc.), consultant will be unavailable. Upon successful completion of pericardiocentesis, participants should be prompted for disposition (if not already determined previously) and END CASE.	BP 110/65 HR 100 RR 16 O2 100% RA Temp 98.6F°
6:00 (**Branch Point 2**: Decompensation)	IV fluid bolus should be ordered before 8:00.	Patient should prompt participant with statements, “I feel terrible,” “I’m so dizzy,” “I’m very weak.” Patient should ask what’s going on, if the cardiologist messed up, and if he should sue the cardiologist. If IV bolus is ordered, case will revert to **Branch Point 1:** Improvement. Vitals will improve. If IV bolus is not ordered by 7:00, nursing will ask the team leader what they think about the low blood pressure reading. If IV bolus is not ordered by 8:00, case will move to **Branch Point 2a:** PEA arrest	BP 70/45 HR 140 RR 30 O2 96% RA Temp 98.6F° With IV fluids BP 110/65 HR 100 RR 16 O2 100% RA Temp 98.6F°
8:00 (**Branch Point 2a**: PEA)	Participants should recognize PEA arrest and initiate CPR.	If learners do poor quality chest compressions, keep end tidal carbon dioxide (ETCO2) at 5, if ETCO2 ordered. If chest compressions are 100–120 bpm and of adequate depth, ETCO2 should be 15 if ETCO2 ordered. If participants order bedside ultrasound during code (if they haven’t already) and successfully perform pericardiocentesis, ROSC will occur and participants will be prompted for disposition and END CASE. If unable to diagnose cardiac tamponade or successfully perform pericardiocentesis, ROSC will not occur, regardless of other interventions performed per advanced cardiac life support (ACLS). END CASE	Pulseless without respirations. PEA is represented as sinus tachycardia 140 BPM on automated external defibrillator (AED). O2 not picking up ETCO2 5–15 If ROSC obtained: BP 100/60 HR 120 RR 16 ETCO2 35 O2 95% RA Temp 98.6F°

**Table:** 

**Critical Actions:** Obtain ECG Administer IV fluid bolus Obtain bedside ultrasound and recognize tamponade physiology Perform bedside pericardiocentesis Admit to a critical care bed.	0:00

**Table:** 

	Milestone	Did not achieve level 1	Level 1	Level 2	Level 3
1	**Emergency Stabilization (PC1)**	□ Did not achieve Level 1	□ Recognizes abnormal vital signs	□ Recognizes an unstable patient, requiring intervention Performs primary assessment Discerns data to formulate a diagnostic impression/plan	□ Manages and prioritizes critical actions in a critically ill patient Reassesses after implementing a stabilizing intervention
2	**Performance of focused history and physical (PC2)**	□ Did not achieve Level 1	□ Performs a reliable, comprehensive history and physical exam	□ Performs and communicates a focused history and physical exam based on chief complaint and urgent issues	□ Prioritizes essential components of history and physical exam given dynamic circumstances
3	**Diagnostic studies (PC3)**	□ Did not achieve Level 1	□ Determines the necessity of diagnostic studies	□ Orders appropriate diagnostic studies. Performs appropriate bedside diagnostic studies/procedures	□ Prioritizes essential testing Interprets results of diagnostic studies Reviews risks, benefits, contraindications, and alternatives to a diagnostic study or procedure
4	**Diagnosis (PC4)**	□ Did not achieve Level 1	□ Considers a list of potential diagnoses	□ Considers an appropriate list of potential diagnosis May or may not make correct diagnosis	□ Makes the appropriate diagnosis Considers other potential diagnoses, avoiding premature closure
5	**Pharmacotherapy (PC5)**	□ Did not achieve Level 1	□ Asks patient for drug allergies	□ Selects an medication for therapeutic intervention, consider potential adverse effects	□ Selects the most appropriate medication and understands mechanism of action, effect, and potential side effects Considers and recognizes drug-drug interactions
6	**Observation and reassessment (PC6)**	□ Did not achieve Level 1	□ Reevaluates patient at least one time during case	□ Reevaluates patient after most therapeutic interventions	□ Consistently evaluates the effectiveness of therapies at appropriate intervals
7	**Disposition (PC7)**	□ Did not achieve Level 1	□ Appropriately selects whether to admit or discharge the patient	□ Appropriately selects whether to admit or discharge Involves the expertise of some of the appropriate specialists	□ Educates the patient appropriately about their disposition Assigns patient to an appropriate level of care (ICU/Tele/Floor) Involves expertise of all appropriate specialists
9	**General Approach to Procedures (PC9)**	□ Did not achieve Level 1	□ Identifies pertinent anatomy and physiology for a procedure Uses appropriate Universal Precautions	□ Obtains informed consent Knows indications, contraindications, anatomic landmarks, equipment, anesthetic and procedural technique, and potential complications for common ED procedures	□ Determines a back-up strategy if initial attempts are unsuccessful Correctly interprets results of diagnostic procedure
20	**Professional Values (PROF1)**	□ Did not achieve Level 1	□ Demonstrates caring, honest behavior	□ Exhibits compassion, respect, sensitivity and responsiveness	□ Develops alternative care plans when patients’ personal beliefs and decisions preclude standard care
22	**Patient centered communication (ICS1)**	□ Did not achieve level 1	□ Establishes rapport and demonstrates empathy to patient (and family) Listens effectively	□ Elicits patient’s reason for seeking health care	□ Manages patient expectations in a manner that minimizes potential for stress, conflict, and misunderstanding. Effectively communicates with vulnerable populations, (at risk patients and families)
23	**Team management (ICS2)**	□ Did not achieve level 1	□ Recognizes other members of the patient care team during case (nurse, techs)	□ Communicates pertinent information to other healthcare colleagues	□ Communicates a clear, succinct, and appropriate handoff with specialists and other colleagues Communicates effectively with ancillary staff

## INSTRUCTOR MATERIALS

**Case Title:** Cardiac Tamponade

**Case Description & Diagnosis (short synopsis):** Patient is a 50-year-old male who presents with chief complaint of dizziness in the setting of a recent ablation procedure for atrial fibrillation. He is anticoagulated on warfarin. Initial vitals will be notable for tachycardia and hypotension which will continue to decompensate to pulseless electrical arrest (PEA) if providers do not quickly perform bedside pericardiocentesis.

### Equipment or Props Needed

High-fidelity human mannequin

Cardiac monitor

Pulse oximetry

Intravenous (IV) pole

Normal saline (1L ×2)

Defibrillator cart

Pericardiocentesis task trainer (such as TraumaMan^®^ or Blue Phantom™’s pericardiocentesis task trainer. Low-cost task trainer options were also described by other authors).^6^,^7^

Pericardiocentesis equipment (30cc syringe, 3.5-inch spinal needle or angiocatheter)

Ultrasound machine

Arterial line task trainer

Medications with labeling: IV vitamin K, prothrombin complex concentrate (PCC), fresh frozen plasma (FFP)

### Confederates needed

Primary nurse

### Stimulus Inventory

**Background and brief information:** Patient is a 50-year-old male who presents with chief complaint of dizziness.

**Initial presentation:** Patient is a 50-year-old male who appears his stated age. He does not appear to be in cardiopulmonary distress and he is answering questions appropriately.

**How the scene unfolds:** Patient will report symptoms of dizziness. When asked to clarify further, patient will describe “feeling like I’m going to pass out” and deny any vertiginous sensation. Patient will further describe acute onset of lightheadedness earlier in the day that occurred at rest and worsens with standing. If asked about associated symptoms, patient will report shortness of breath that is worsened by exertion, but deny any other complaints, including chest pain, palpitations, or syncope. Vitals are notable for tachycardia with mild hypotension.

If patient is given resuscitative fluid boluses, his hypotension will improve transiently, but the hypotension will ultimately worsen. If learners attempt to contact cardiology, the cardiologist will be busy in the catheterization lab, prompting learners to perform bedside pericardiocentesis.

The patient will ask learners what is going on and if this is the cardiologist’s fault. The patient will then ask the learners if he should sue the cardiologist.

If patient is not promptly (less than 5min) given resuscitative fluids, patient will begin to decompensate with worsening hypotension and increasing tachycardia until patient is given IV fluids. If IV fluids continue to be withheld and a pericardiocentesis is not performed in a timely fashion, patient will go into PEA arrest. Return of spontaneous circulation (ROSC) is then at the discretion of the facilitator, but ROSC may occur after pericardiocentesis is successfully performed.

### Critical actions

1. Obtain ECG
2. Administer IV fluid bolus
3. Obtain bedside ultrasound and recognize tamponade physiology
4. Perform bedside pericardiocentesis
5. Admit to a critical care bed

**Case Title:** Cardiac Tamponade

**Chief Complaint:** Dizziness

**General Appearance:** Alert, conversational, no acute cardiopulmonary distress

### Primary Survey

- **Airway:** patent
- **Breathing:** clear to auscultation bilaterally
- **Circulation:** Tachycardic, 2+ symmetric radial pulses bilaterally, capillary refill 2–3 seconds

### History

- **History of present illness:** Patient reports sudden onset of dizziness which began 6 hours ago at rest. When asked, he describes it as feeling “like I’m going to pass out” and worsens with standing. He also reports associated shortness of breath, worsening with exertion.

He denies fever, tinnitus, upper respiratory infection symptoms, headache, chest pain, palpitations, nausea, vomiting, diarrhea, melena, hematochezia, urinary complaints, wounds or sores, unilateral weakness or numbness, trouble speaking or swallowing, facial droop.

If asked, he has had a recent ablation procedure 2 days ago for symptomatic atrial fibrillation refractory to rate control. He has continued his warfarin and has been compliant with this. Denies any other medication changes.

- **Past medical history**: atrial fibrillation, hypertension, and obstructive sleep apnea
- **Past surgical history**: Ablation for atrial fibrillation
- **Medications**: amlodipine, warfarin
- **Allergies**: no known drug allergies
- **Social history**: No tobacco, alcohol, or illicit substance use
- **Family history**: unknown

### Secondary Survey/Physical Examination

**General appearance:** Alert, conversational, no acute cardiopulmonary distress

- **HEENT:**
  ○ **Head:** within normal limits
  ○ **Eyes:** within normal limits
  ○ **Ears:** within normal limits
  ○ **Nose:** within normal limits
  ○ **Throat:** within normal limits
- **Neck:** jugular venous distention (JVD) (if asked)
- **Heart:** Regular tachycardic rhythm, 2+ symmetric distal pulses. Otherwise within normal limits
- **Lungs:** Mild conversational dyspnea. Clear breath sounds. Otherwise within normal limits
- **Abdominal/GI:** within normal limits
- **Genitourinary:** within normal limits
- **Rectal:** within normal limits
- **Extremities:** within normal limits
- **Back:** within normal limits
- **Neuro:** within normal limits
- **Skin:** within normal limits

### Results

*Chest Radiograph (CXR)*

Dilmen N. Medical X-rays. In: Wikimedia Commons.

https://commons.wikimedia.org/wiki/File:Rad_1300124.JPG. Published September 21, 2010. CC BY-SA 3.0.

*Electrocardiogram (EKG)*

CardioNetworks: Vdbilt. Pulsus Alternans. In: Wikimedia Commons.

https://commons.wikimedia.org/wiki/File:PulsusAlternans_(CardioNetworks_ECHOpedia).jpg Published Sept 24, 2007. CC BY-SA 3.0.

*Pericardial effusion*

Smith B. Ultrasound of the week. In: Wikimedia Commons.

https://commons.wikimedia.org/wiki/File:UOTW_78_-_Ultrasound_of_the_Week_4.jpg. Published February 27, 2017.

*Plethroic IVC*

Dilmen, N. Ultrasound scan. In: Wikimedia Commons.

https://commons.wikimedia.org/wiki/File:Ultrasound_Scan_ND_0104134930_1355510.png. Published 2011. CC BY-SA 4.0.

## OPERATOR MATERIALS

### Diagnosis

Cardiac tamponade

### Disposition

Admission to the intensive care unit

## DEBRIEFING AND EVALUATION PEARLS

### Cardiac Tamponade

- *Pathophysiology*: Cardiac tamponade is a rare life-threatening condition evolving from a fluid collection within the pericardial sac. As fluid accumulates within this closed space, the pressure exerted by the fluid increases. When this pressure becomes greater than the filling pressures within the chambers of the heart (~5 mmHg for right ventricle, ~10 mmHg for left ventricle for an average adult individual), filling becomes compromised, resulting in obstructive shock and hemodynamic collapse.^1^,^2^,^4^
- *Etiology*: The causes of cardiac tamponade are numerous; any condition that may lead to development of pericardial effusions (infection, malignancy, recent MI, autoimmune disorders, etc.) can also theoretically result in cardiac tamponade. However, certain etiologies of pericardial effusions which lead to a more rapid rate of fluid accumulation (ie, hemorrhagic) are more likely to induce tamponade physiology.^1^,^2^,^8^
- *Pulsus paradoxus:* Inflate a blood pressure cuff on the patient’s arm. As you deflate the cuff, have the patient exhale. Record the first Korotkoff sound. Repeat the measurement, but have the patient inhale while auscultating. If the difference between the two sounds is greater than 10mmHg, it may be classified as pulsus paradoxus.
- *Diagnostic testing*: Because cardiac tamponade is a form of obstructive shock, patients with this condition typically present with symptoms consistent with shock, such as lightheadedness, as well as symptoms of venous pooling of fluid secondary to the obstruction, including shortness of breath, chest discomfort, leg edema, etc. In addition, patients may present with vital sign abnormalities such as tachycardia and hypotension. As a result of this non-specific presentation, the initial diagnostic workup for these patients will need to be broad to include a multitude of common etiologies of shock (ie, sepsis, myocardial infarction, hemorrhage, pulmonary embolism, etc.). The definitive diagnosis of cardiac tamponade can be made using ultrasound, although less sensitive findings sometimes seen on ECG can be suggestive and should raise the index of suspicion for this relatively rare condition.^10^–^11^
- *ECG findings*: A number of ECG findings have been associated with cardiac tamponade, including low voltage QRS complexes, PR segment depression, and electrical alternans. Although research in this area is somewhat limited by the rarity of this condition, general consensus is that these findings are poorly sensitive for the condition but are relatively specific and can be suggestive of the diagnosis if observed.^12^–^16^
- *Echocardiography findings*: While the definitive diagnosis of cardiac tamponade was previously diagnosed using pulsus paradoxus, the increasing availability and sophistication of point-of-care ultrasound has greatly improved the emergency physician’s ability to diagnose this condition in an efficient and accurate manner at bedside. The findings typically seen on ultrasound correlating with cardiac tamponade include systolic right atrial (RA) collapse (earliest finding), diastolic right ventricle (RV) collapse, plethoric inferior vena cava (IVC) with minimal respiratory variation, as well as mitral valve inflow variation. While a pericardial effusion, typically with circumferential involvement, is a pre-requisite to a diagnosis of tamponade, the size of the effusion itself cannot be correlated to the presence of acute tamponade physiology.^10^
- *Pericardiocentesis Technique*: Pericardiocentesis has traditionally been taught as a blind subxiphoid approach. More recently, support for more novel ultrasound-guided approaches (ie, parasternal) is on the rise, but the evidence for these approaches in an emergency department setting is limited. In this case, a traditional subxiphoid approach was reviewed during debriefing, although learners were also encouraged to use ultrasound guidance for a subxiphoid approach:^17^–^19^

1. Palpate the xyphoid process. Move 2 fingerbreadths inferior to this and then 2 fingerbreadths laterally to the left (just below the costal margin).
2. Insert needle at 30 degrees, directed towards the left shoulder. Aspirate as the needle is advanced. Ultrasound may be used for ultrasound guidance, if available.
3. When blood return is obtained, remove up to 30 cc of fluid. If ultrasound is available, you may inject fluid back in to confirm location of the needle tip. If the needle is appropriately positioned, you should visualize turbulence in the pericardial fluid and not within the ventricles.
4. Continue to remove pericardial fluid until patient’s vital signs improve (usually requires approximately 30cc, but more may be needed).

### Additional Debriefing Points

- When asking patients to elaborate on their dizziness, the authors prefer to ask the patient to “Describe your dizziness without using the word ‘dizzy.’” This may help distinguish vertiginous symptoms from presyncope. Further questioning may reveal if there were any associated symptoms, such as chest discomfort, dyspnea, vision change, or palpitations. While vertigo may lead down a cerebellar pathology diagnostic pathway, presyncope isn’t without underlying harmful etiologies. It is hypothesized it has a similar underlying cause of global cerebral hypoperfusion as syncope,^21^ and presyncope was associated with a significant number of adverse outcomes.^22^
- *Disclosing Medical Errors*: In this simulation case, there was a unique debriefing point regarding the process of medical error disclosure, given the iatrogenic etiology of the patient’s condition. This raised an interesting discussion regarding whether a medical error should be disclosed to patients and what the appropriate course of action should be regarding disclosure, if indicated. The general consensus regarding this issue suggests that while disclosure is generally the ethical course of action, the process of disclosure, if done improperly, can put a physician in medico-legal risk. The laws surrounding the process of disclosure differ state by state, and it is important to understand the legal environment in which one practices. In California and Florida, disclosure of medical errors is mandated by law. A large number of states have apology statutes, which hold a provider’s medical error disclosure and apology inadmissible in court, although the nuances surrounding these statutes vary state by state.^22^ For example, in the Ohio appellate court system, a case was ruled in favor of the plaintiff after an orthopedic surgeon had verbally admitted fault to the plaintiff, stating that the law’s “intent was to protect pure expressions of apology, sympathy, commiseration, condolence, compassion, or a general sense of benevolence, but not admissions of fault.” 23 Because of the medico-legal implications of disclosing medical errors, it is generally considered prudent to first discuss cases with your institution’s medical-legal team or to consult institution-specific policy.^24^

## SIMULATION ASSESSMENT

*Cardiac Tamponade*

Learner: _________________________________________

***Assessment Timeline***

This timeline is to help observers assess their learners. It allows observer to make notes on when learners performed various tasks, which can help guide debriefing discussion.

**Critical Actions:**

□ Obtain ECG
□ Administer IV fluid bolus
□ Obtain bedside ultrasound and recognize tamponade physiology
□ Perform bedside pericardiocentesis
□ Admit to a critical care bed.

**Summative and formative comments:**

**Milestones assessment:**
