# Supplementary material for: Cardiac Tamponade
Source: J Educ Teach Emerg Med. 2020 Oct 15;5(4):S84–S107. doi: 10.21980/J81D1D (PMC10332517; doi:10.21980/J81D1D)
Supplement: Supplementary file 1 [file jetem-5-4-s84-supp1.pptx]

## Slide 1
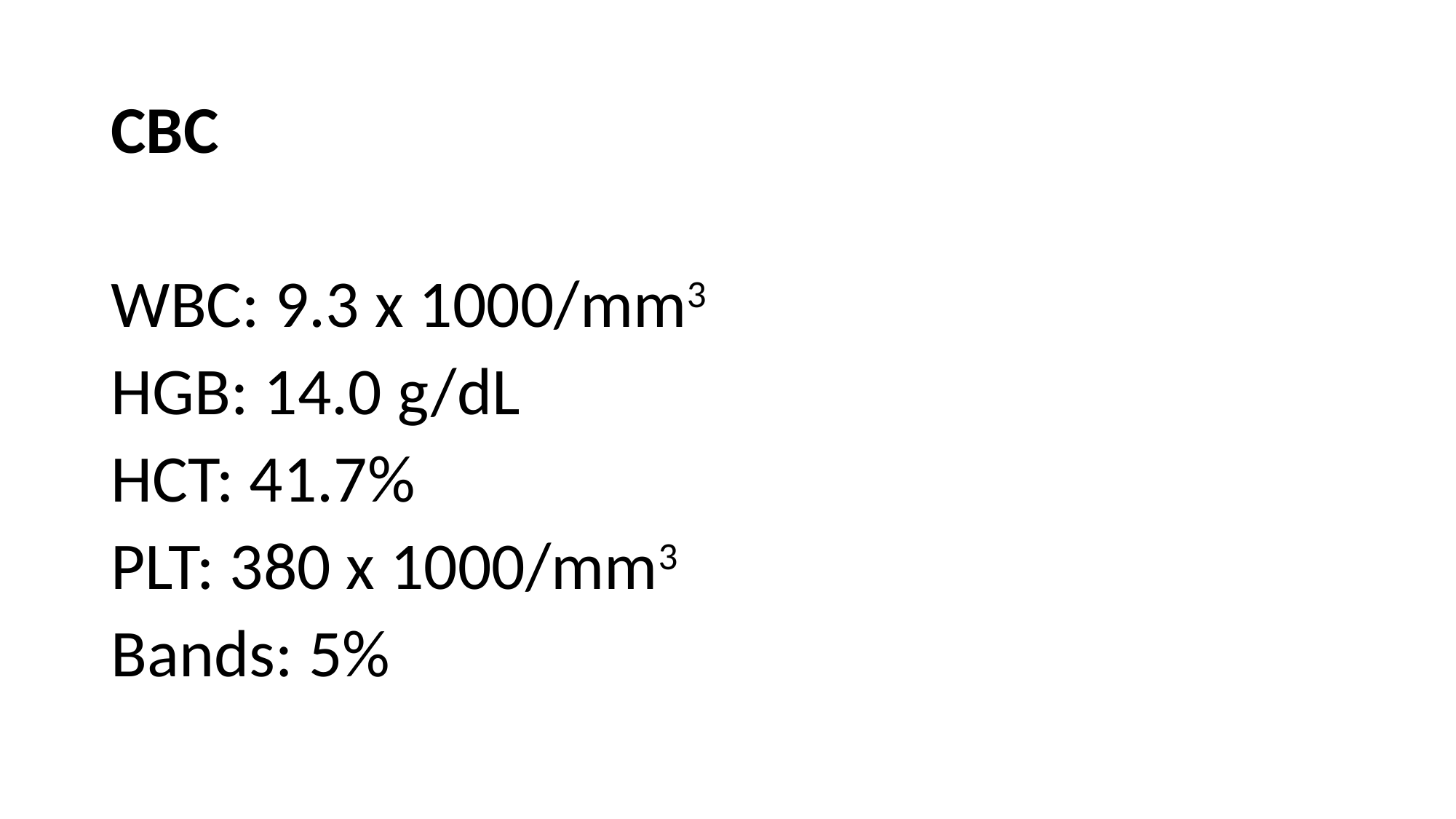

CBC
WBC: 9.3 x 1000/mm3
HGB: 14.0 g/dL
HCT: 41.7%
PLT: 380 x 1000/mm3
Bands: 5%

## Slide 2
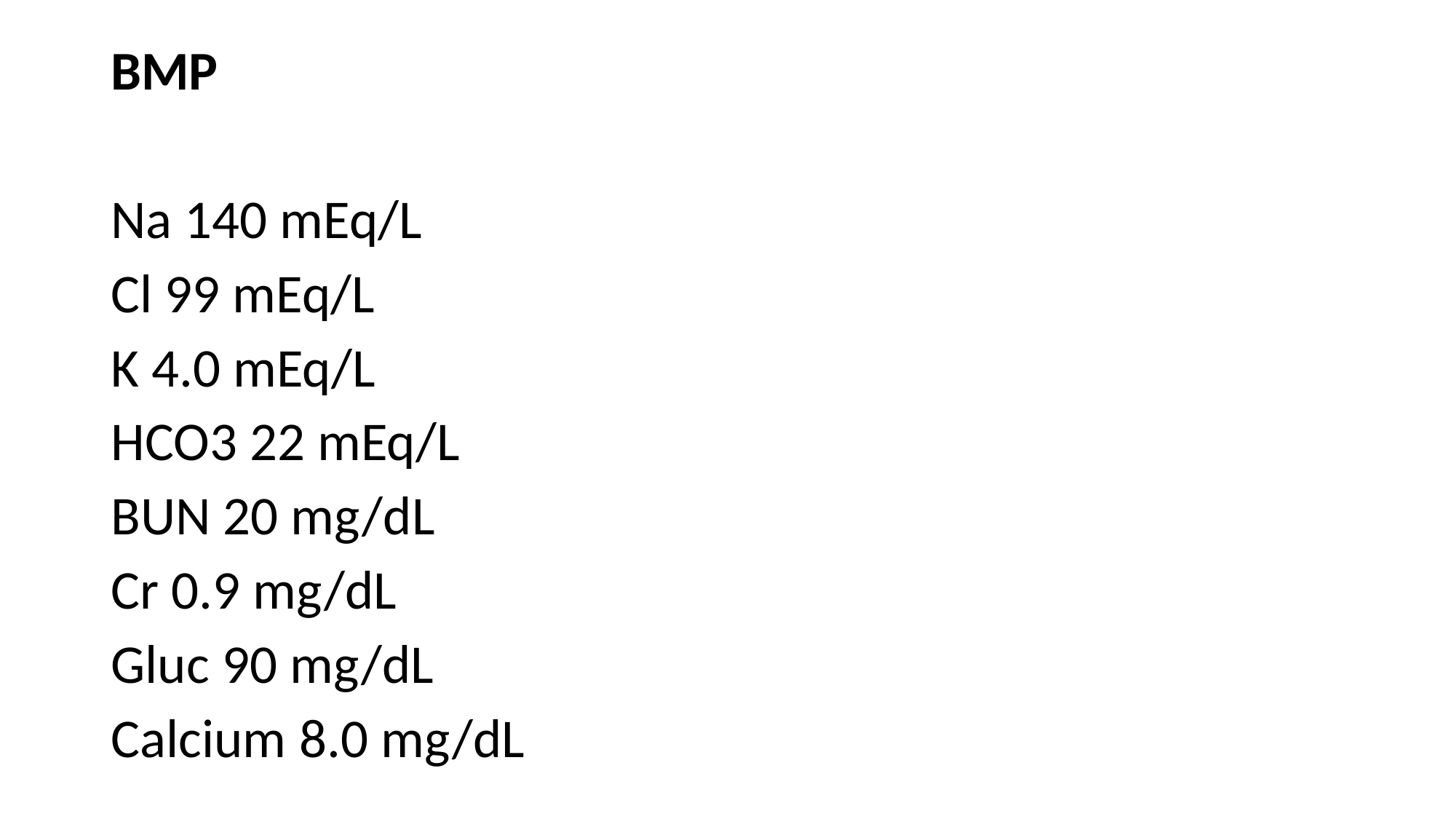

BMP
Na 140 mEq/L
Cl 99 mEq/L
K 4.0 mEq/L
HCO3 22 mEq/L
BUN 20 mg/dL
Cr 0.9 mg/dL
Gluc 90 mg/dL
Calcium 8.0 mg/dL

## Slide 3
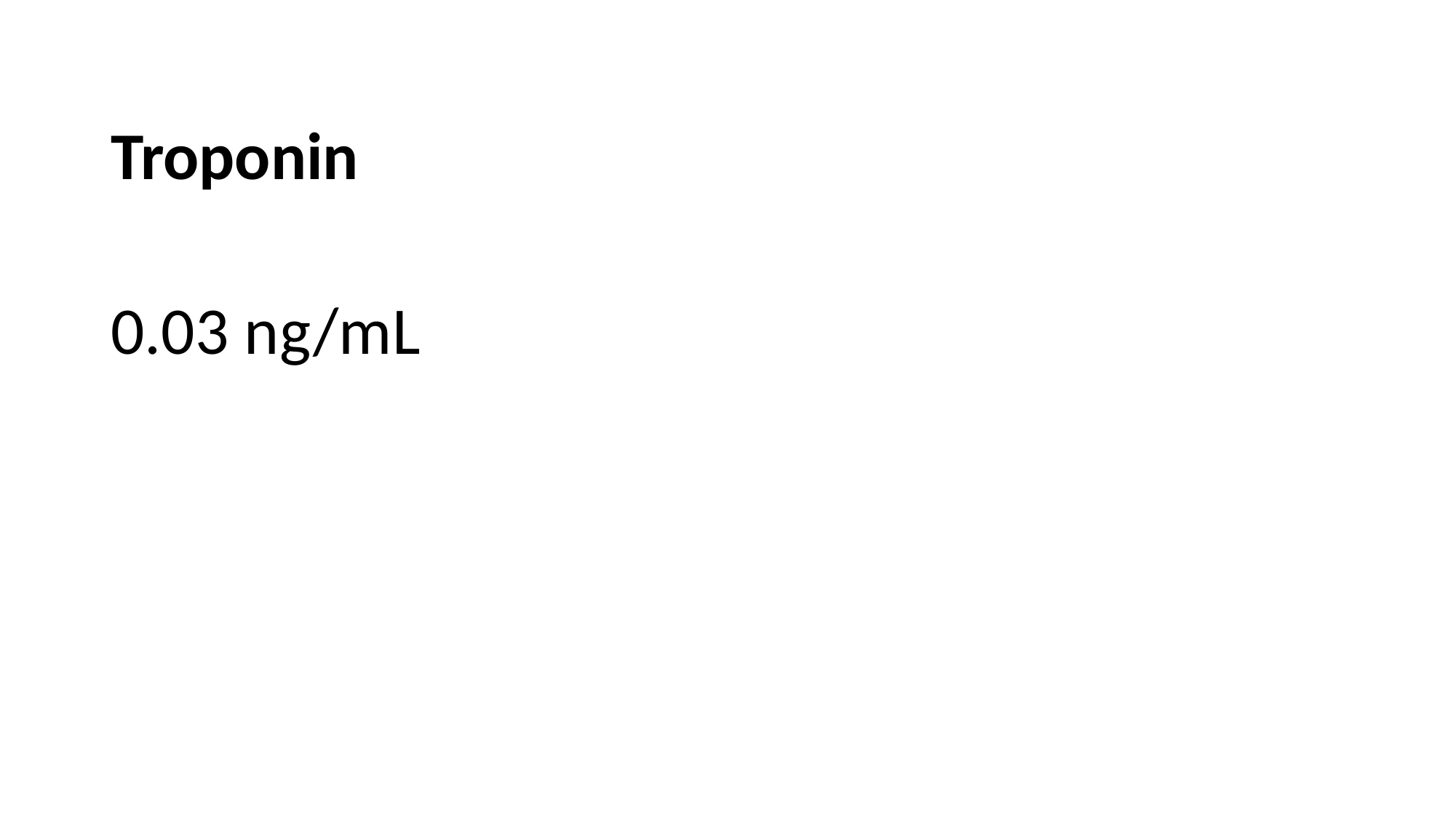

Troponin
0.03 ng/mL

## Slide 4
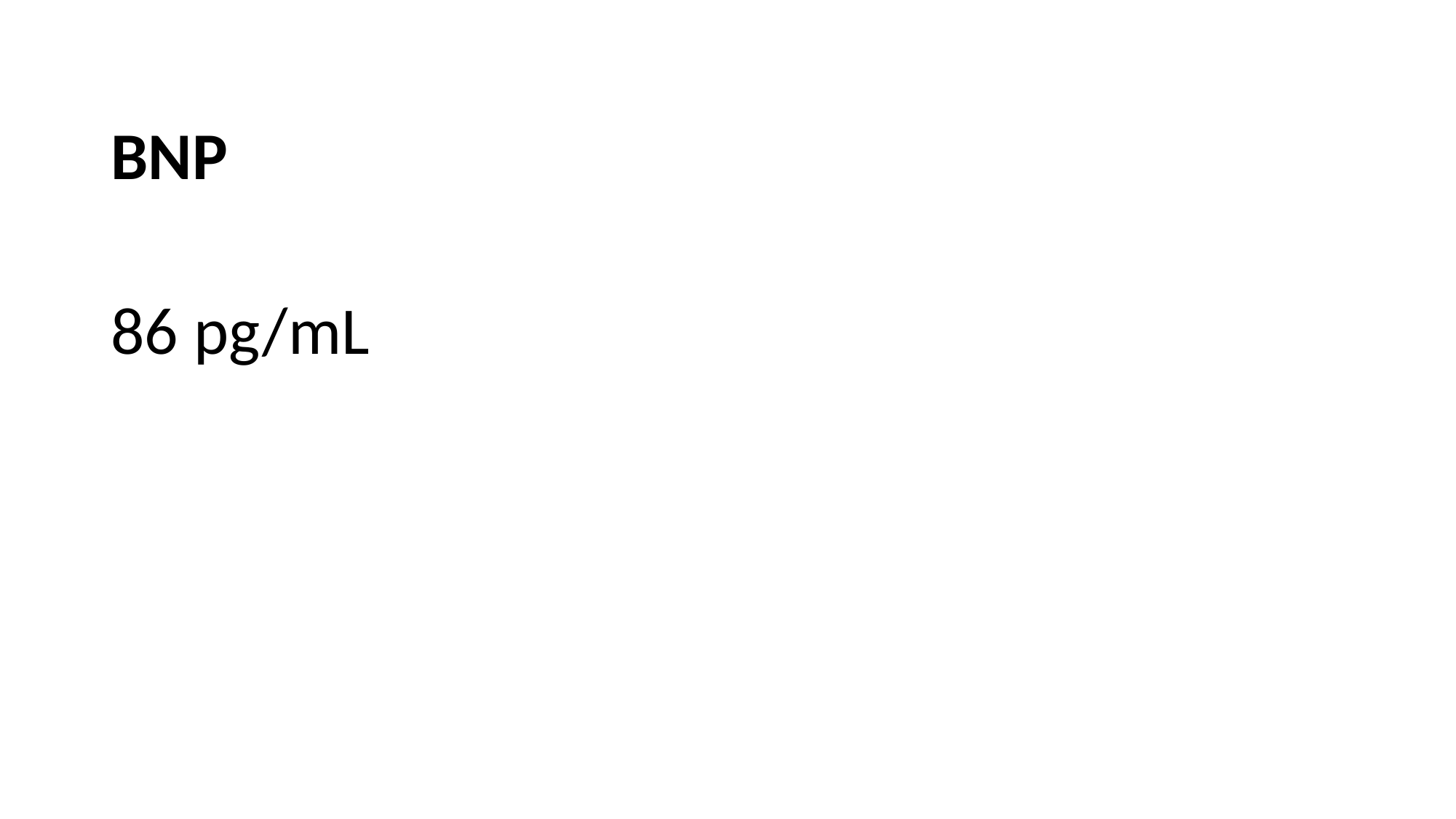

BNP
86 pg/mL

## Slide 5
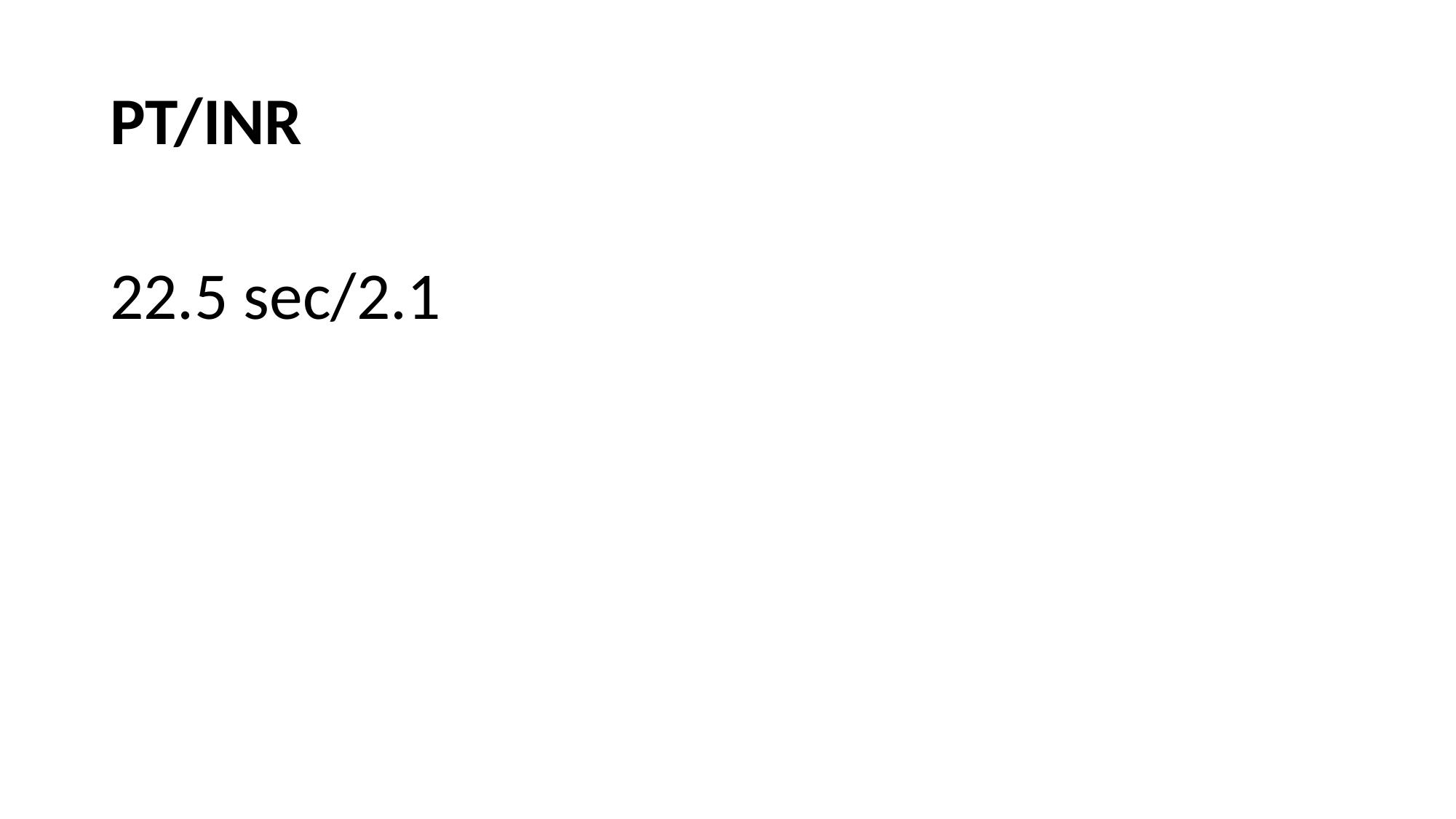

PT/INR
22.5 sec/2.1

## Slide 6
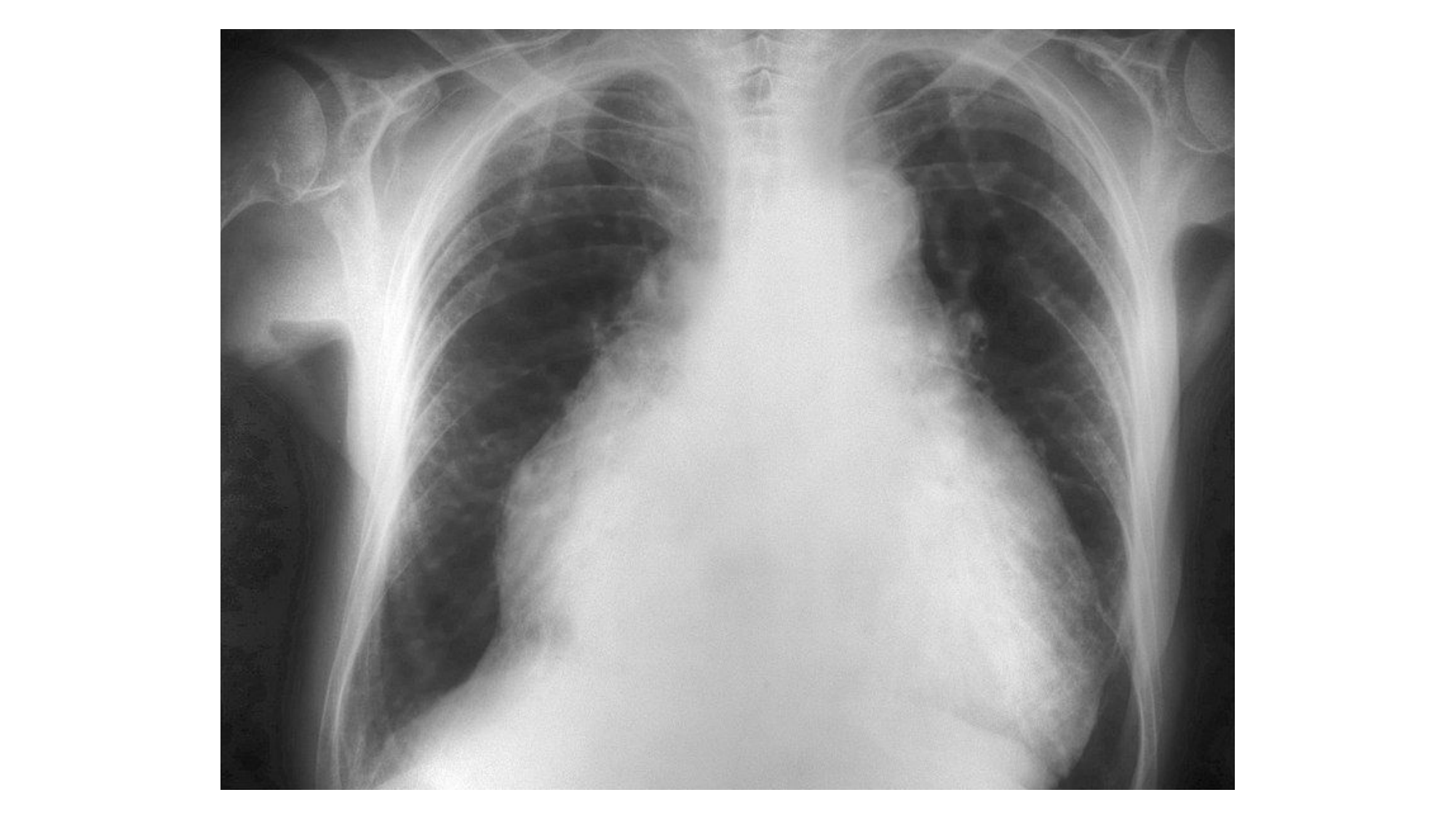

## Slide 7
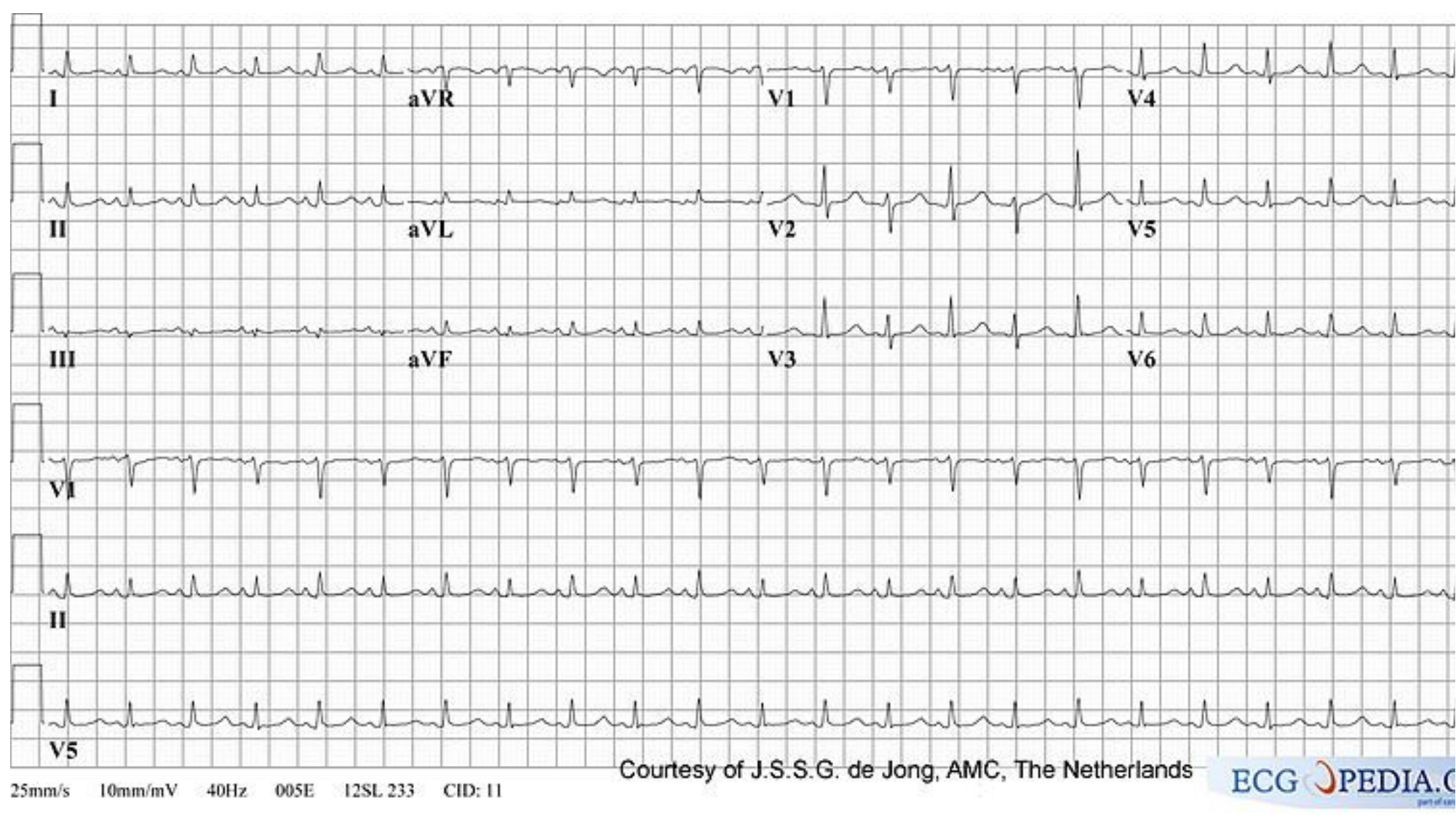

Lwo voltage ecg

## Slide 8
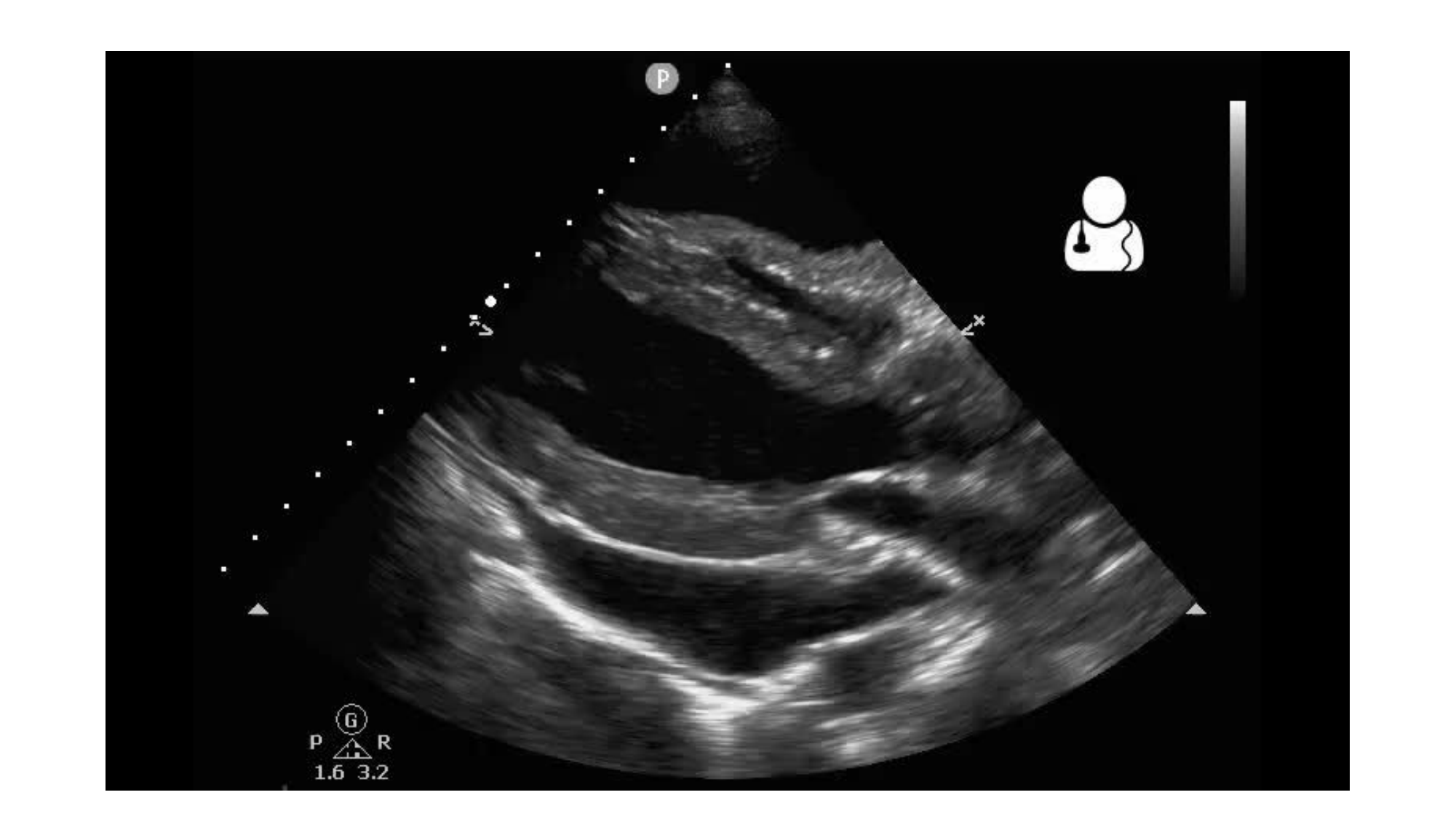

## Slide 9
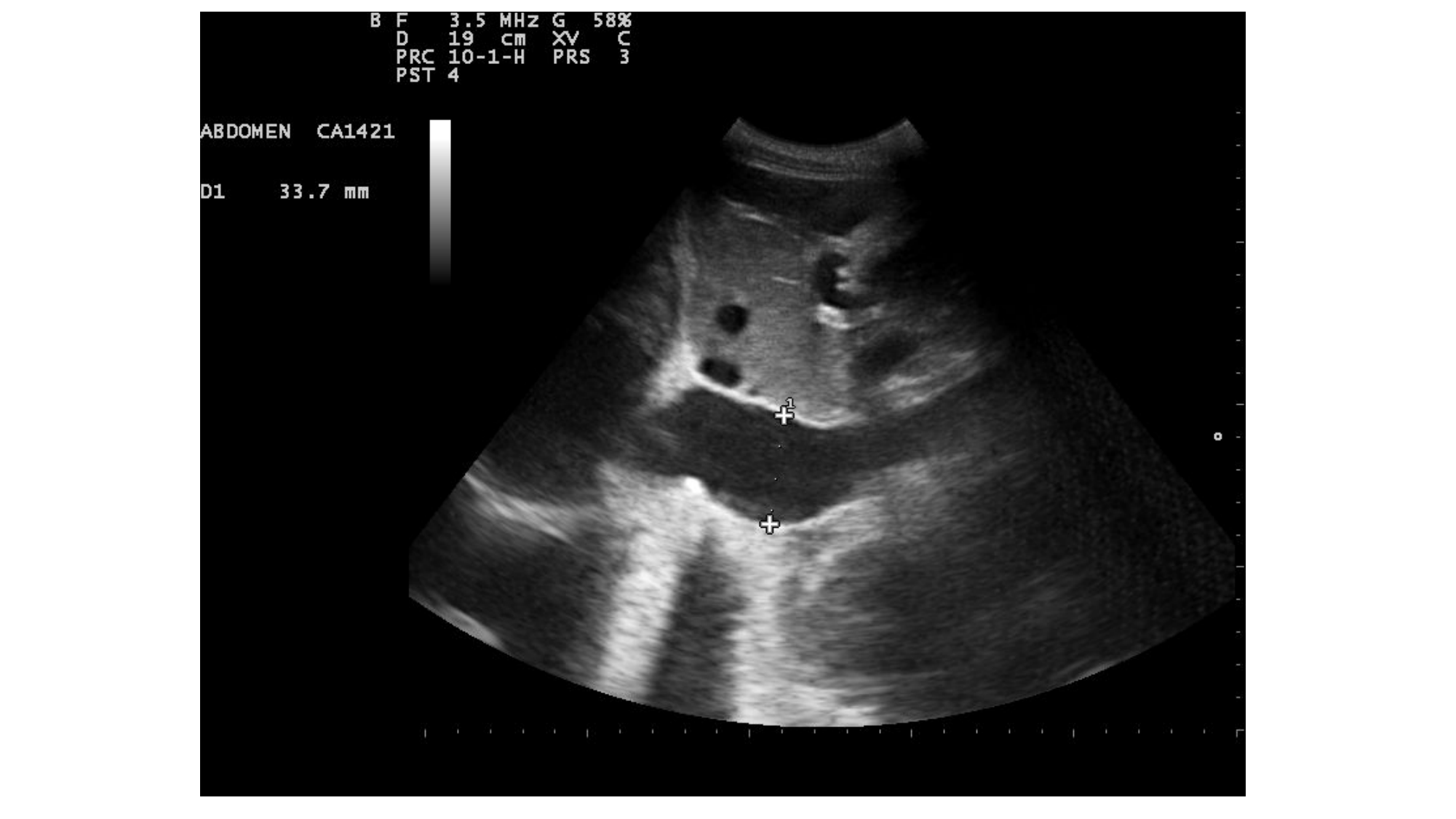

#
